# The experience of dry mouth and screening for Sjogren’s syndrome by the dentist: patient-reported experiences

**DOI:** 10.1186/s12903-023-03727-z

**Published:** 2023-12-15

**Authors:** Boughanmi Rihab, El Houari Lina, Simon-Tillaux Noémie, Saide Jean, Gosset Marjolaine

**Affiliations:** 1 Service de Médecine Bucco-Dentaire, AP-HP, Hôpital Charles Foix, Ivry/seine, F-94200 France; 2 Faculté de Médecine Dentaire, Université de Monastir, LR12ES11, Monastir, 5000 Tunisia; 3 AP-HP, Hôpital Pitié Salpêtrière, Département de Santé Publique, Centre de Pharmacoépidémiologie (Cephepi), Unité de Recherche Clinique PSL-CFX, CIC-1901, Paris, F75013 France; 4 Département de Santé Publique, Centre de Pharmacoépidémiologie (Cephepi), Unité de Recherche Clinique PSL-CFX, Sorbonne Université, INSERM, Institut Pierre Louis d’Épidémiologie et de Santé Publique, AP-HP, Hôpital Pitié Salpêtrière, CIC-1901, Paris, F75013 France; 5 Association Française pour les Patients atteints de Gougerot Sjögren et des Syndromes Secs, Paris, F- 75018 France; 6 Université Paris Cité, URP 2496, 1 rue Maurice Arnoux, Montrouge, F-92120 France; 7 Laboratoire d’Excellence INFLAMEX, Paris, France

**Keywords:** Dry mouth, Sjögren’s syndrome, Patient association, Dentist, Patient experience

## Abstract

**Background:**

One of the main clinical features of Sjögren’s Syndrome is oral dryness, which is associated with an increased risk of oral diseases and a lower oral life quality. Dentists have a key role to play in the Sjögren’s Syndrome diagnosis and specific management. In parallel, many patients rely on patient associations, which offer opportunities for members to seek information about their disease and share their experiences. We aimed to evaluate patients experience with dry mouth and the importance of dentists in Sjögren’s Syndrome diagnosis and its management.

**Methods:**

We carried out a cross-sectional survey in 2020 based on a questionnaire drafted in collaboration with clinicians specializing in Sjögren’s Syndrome and patient members of a patient association. The survey consisted of 27 questions divided into the six sections: the patient’s profile*,* their experience with dry mouth and treatments used to manage, characteristics of experienced oral-health problems*,* effects of dry mouth and its consequences on the quality of life*,* evaluation of the dentist role in the screening of Sjögren’s Syndrome, and its management by the dentist. Recruitment was carried out via the patient association’s newsletter, website, and social networks. Sjögren’s diagnosis was self-reported.

**Results:**

One thousand four hundred fifty-eight patients fully responded to the survey. Most respondents were women over 50 and were mainly concerned with primary Sjögren’s Syndrome. Overall, 86.97% of respondents reported experiencing frequent or constant dry mouth and 69.01% declared having had oral problems (candidiasis, oral pain, loss or alteration of taste, bad breath, gastro-esophageal reflux). We found a positive correlation between the frequency of dry mouth and each of these disorders and between the frequency of dry mouth and alterations in life quality dimensions. Finally, 74.9% of patients did not report having dry mouth to their dentist prior to being diagnosed with Sjögren’s Syndrome and 58% had not been informed about the oral risks associated with it by their dentist and sought information themselves or from their physician.

**Conclusions:**

We confirm the significant consequences of dry mouth on oral quality of life, as well as its association with oral health problems. Sjögren’s Syndrome screening by dentists should be increased, as well as prevention of the associated oral health risks.

**Supplementary Information:**

The online version contains supplementary material available at 10.1186/s12903-023-03727-z.

## Introduction

Primary Sjögren’s syndrome is a rare systemic autoimmune disease characterized by impairment of the secretory functions of the exocrine glands [1]. It is mainly characterized by the association of a triad of symptoms (dryness, pain, and asthenia), of which ocular and oral dryness are associated with a deterioration in the quality of life [2, 3]. Sjogren’s syndrome is said to be “associated” when it is associated with other systemic autoimmune diseases (such as rheumatoid arthritis, systemic lupus erythematosus, or systemic sclerosis). Finally, dryness may exist without a diagnosis of Sjögren’s syndrome. It effects around 22% of the population, with an increase in prevalence according to age [4].

Dry mouth represents a true issue in terms of oral health: hyposialia is indeed associated with an increased risk of oral diseases (candidiasis, dental caries, dental erosion) [3] and a lower oral quality of life [5]. The dentist therefore has a key role to play in the detection of dry mouth and primary Sjögren’s syndrome and implementation of adapted prevention of oral risks in these patients. However, there is little information in the literature on the role of dentists, the main providers of oral health care, in the diagnosis and management of these patients.

The impact of primary Sjögren’s syndrome on patients’ quality of life has been explored in quantitative studies using questionnaires, such as the SF-36 (The short Form Health Survey in 36 items), assessing the general health-related quality of life, and the OHIP-14 or − 49 (Oral Health Impact Profile in 14 or 49 items), assessing the oral health-related quality of life. These studies showed deterioration in the health-related quality of life for patients with oral dryness [2]. Qualitative studies based on the analysis of structured interviews have provided additional information by exploring the experience of patients with primary Sjögren’s disease. They highlight the physical consequences of dry mouth already described in the literature (difficulty in moving the lips and mouth, partial or total loss of taste and smell, difficulty in eating, swallowing, and speaking), without succeeding in measuring the impact of dry mouth on the quality of life of the patients, the symptoms being experienced as an inseparable whole [6–8].

Here, we aimed to evaluate the experience of patients with dry mouth in the context of dry mouth syndrome and the importance of dentists in the diagnosis and specific management of dry mouth of patients with Sjögren’s disease through a survey co-constructed with the French Patient Association (Association Française du Gougerot-Sjögren et des Syndromes Secs, AFGS).

## Methods

### Design of the study

A collaboration was established between Professor Marjolaine Gosset (MG), who runs a specialized oral medicine consultation at the Charles Foix Hospital for patients suffering from dry mouth, and the French Association of Gougerot Sjögren and Dry Syndromes (AFGS) to conduct a national cross-sectional survey among members of the AFGS.

### Study population

The survey was distributed by the AFGS to its members (2400 in 2020, 96% of whom said they had Sjögren’s or Sicca’s syndrome). It was also available to non-members. To answer the questionnaire, people had to declare that they had primary Sjögren’s, secondary Sjögren’s, or Sicca syndrome (no other choice possible).

### Data collection

A semi-structured questionnaire containing 27 questions was developed by MG and five patients from the AFGS. The questionnaire was divided into six sections, as follows: 1) the patient’s profile (gender, age, medical diagnosis, most disabling symptom); 2) the participant’s experience of dry mouth and treatments used to manage it (intensity of dryness, use of pilocarpine or local dry mouth devices, effectiveness of treatment); 3) the characteristics of experienced oral health problems (caries, tooth wear, gum disease, dental care failures, difficulties in wearing removable prostheses, dental implant failures, mouth ulcers, candidiasis, oral pain, taste alterations, halitosis, gastro-esophageal reflux); 4) the effects of dry mouth and its consequences on quality of life (discomfort, quality of sleep, discomfort in social life, concern about the evolution of the mouth, concern about the costs of care); 5) an evaluation of the role of the dentist in the screening of Sjogren’s Syndrome (diagnostic approach following the declaration of a dry mouth by the patient, severity of oral damage as a warning sign); and 6) the management of Sjögren’s syndrome by the dentist (information on the consequences of dryness on oral health, prescription of adequate materials and products, follow-up advice).

The questionnaire was tested by 15 patients from the AFGS. Their feedback on the comprehension of the questions and the ease of answering them helped us to improve and finalize the questionnaire. The questionnaire took approximately 15 min to complete. The questionnaire was distributed on a secure platform provided by the University of Paris (Lime Survey®), allowing anonymous online completion, storage of the answers, and data extraction. AFGS members were invited to complete the questionnaire via the AFGS newsletter, website, and social networks. The questionnaire was available on the website of the AFGS between January 9 and March 2, 2020 (Additional file 1).

### Statistical analysis

Qualitative variables are presented as numbers and percentages and quantitative variables as numbers, means, and standard deviations. Categorical variables were compared using Chi-squared or Fisher tests, as appropriate. Continuous endpoints were compared using Student’s t test. To assess the dose-response relationship between the frequency of dry mouth and other symptoms, trend tests (Cochran Armitage Chi-squared tests) were used to compare the proportion of participants with symptoms between the ordinal categories of frequency of experiencing dry mouth (never, sometimes, quite often, always). A significant *p*-value for a trend indicates that the proportion of participants with each symptom significantly increases if the frequency of the experience of dry mouth increases.

Statistical analyses were performed using STATA software (Stata Statistical Software: Release 15. College Station, TX: StataCorp LLC) and R version 4.2 [9]. All tests were two-sided and a *p* value < 0.05 was the threshold for statistical significance.

## Results

### Profile of respondents

In total, 1458 patients fully responded to the survey. As the survey was open to non-member patients, we can only estimate the participation rate using the 2400 AFGS members solicited to participate in the survey as a reference. Therefore, the maximum participation rate was 60.75%. Overall, 96.16% of the respondents were women and 52.3% were over 60 years of age. The respondents declared primary Sjögren’s syndrome (59.12%), an associated autoimmune disease (“associated” Sjögren’s syndrome - 30.4%), or dry syndrome (10.4%) (Table 1).

**Table 1:** Characteristics of the population

	Number (%)
**Sex**	
Female	1402 (96.16)
Male	56 (3.84)
**Age**	
Less than 40 years old	123 (8.4)
40 to 50 years old	226 (15.5)
50 to 60 years old	346 (23.7)
Over 60 years old	763 (52.3)
**Declared pathology**	
Primary Sjögren’s syndrome	862 (59.12)
Associated Sjögren’s syndrome	444 (30.4)
Dry Syndrome	152 (10.4)

Regardless of the nature of the syndrome, dry mouth appeared to be the symptom that most affected the quality of life. A balanced distribution between the four symptoms was observed for patients with primary Sjögren’s syndrome, whereas it was more marked for articular and muscular pain for patients with associated Sjögren’s syndrome. The second symptom reported by patients with dry syndromes was dryness of the eye (Table 2).

**Table 2:** Assessment of the symptoms affecting respondents’ quality of life based on the reported form of the syndrome

		I am affected by			
		Primary Sjögren *N* = 862 (59.12%)	Associated Sjögren *N* = 444 (30.4%)	Dry Syndrome *N* = 152 (10.4%)	*p*-value (Fisher’s Exact Test)
**Of these four symptoms, the one that affects my quality of life the most is.**	Asthenia	213 (24.71%)	108 (24.32%)	28 (18.42%)	< 1e-04
	Mouth Dryness	257 (29.81%)	105 (23.65%)	65 (42.76%)	
	Eye Dryness	175 (20.3%)	80 (18.02%)	39 (25.66%)	
	Articular and Muscular pain	217 (25.17%)	151 (34.01%)	20 (13.16%)	

### Experience of patients with dry mouth

#### Characteristics of reported oral conditions

Among survey respondents, 86.97% reported that their mouth was often or always dry. Among them, 69.01% declared to have oral problems (mainly caries, dental wear, and gum problems) versus 53.68% of patients without or with a low frequency of oral dryness (Table 3). In addition, these patients reported to be more affected by the presence of mouth ulcers, oral candidiasis, oral pain or burning, loss or alteration of taste, bad breath, and gastro-esophageal reflux (Table 3).

**Table 3:** Evaluation of the association between dry mouth and oral disorders. Participants were deemed to have oral dryness if they answered ‘always’ or ‘quite often’ to the question that explored the frequency of experiencing dry mouth

		Without oral dryness (%) *N* = 190	With Oral dryness (%) *N* = 1268	***p***-value
**Oral and dental problems**	Yes	102 (53.68)	875 (69.01)	< 0.0001 (Fisher’s Exact Test)
	No	88 (46.32)	393 (30.99)	
**Mouth ulcers**	Never	75 (39.47)	334 (26.34)	0.0005 (Pearson’s Chi-squared test)
	Sometimes	92 (48.42)	623 (49.13)	
	Quite often	21 (11.05)	278 (21.92)	
	Always	2 (1.05)	33 (2.6)	
**Oral candidiasis**	Never	140 (73.68)	596 (47)	< 0.0001 (Pearson’s Chi-squared test)
	Sometimes	38 (20)	559 (36.2)	
	Quite often	7 (3.68)	172 (13.56)	
	Always	5 (2.63)	41 (3.23)	
**Oral pain or burning**	Never	101 (53.16)	330 (26.03)	< 0.0001 (Pearson’s Chi-squared test)
	Sometimes	62 (32.63)	417 (32.89)	
	Quite often	19 (10)	386 (30.44)	
	Always	8 (4.21)	135 (10.65)	
**Loss or alteration of taste**	Never	84 (44.21)	344 (27.13)	< 0.0001 (Pearson’s Chi-squared test)
	Sometimes	77 (40.53)	482 (33.01)	
	Quite often	23 (12.11)	329 (25.95)	
	Always	6 (3.16)	113 (8.91)	
**Bad mouth breath**	Never	67 (35.26)	328 (25.87)	0.0049 (Pearson’s Chi-squared test)
	Sometimes	91 (47.89)	595 (46.92)	
	Quite often	26 (13.68)	254 (20.03)	
	Always	6 (3.16)	91 (7.18)	
**Gastro-esophageal reflux**	Never	45 (23.68)	254 (20.03)	0.0104 (Pearson’s Chi-squared test)
	Sometimes	79 (41.58)	410 (32.33)	
	Quite often	37 (19.47)	341 (26.89)	
	Always	29 (15.26)	263 (20.74)	

Participants were deemed to have oral dryness if they answered ‘always’ or ‘quite often’ to the question that explored the frequency of experiencing dry mouth

Interestingly, we observed a dose-response relationship between the frequency of experiencing dry mouth and each of these disorders (Table 4): the drier the mouth, the more patients reported frequently experiencing oral problems, mouth ulcers, candidiasis, loss or alteration of taste, bad breath, and gastroesophageal reflux.

**Table 4:** Dose-response relationship between the intensity of experienced dry mouth and oral disorders

	Frequency of dry mouth				***p*** for trend (Cochran Armitage Chi-squared test)
	Never ***N*** = 19	Sometimes ***N*** = 171	Quite often ***N*** = 623	Always ***N*** = 645	
**Oral and dental problems (%, yes)**	8 (42%)	94 (55%)	407 (65%)	468 (73%)	< 0.0001
**Mouth ulcers (%, often or always)**	1 (5%)	22 (13%)	124 (20%)	187 (29%)	< 0.0001
**Oral candidiasis (%, often or always)**	0 (0%)	12 (7%)	64 (10%)	149 (23%)	< 0.0001
**Oral pain or burning (%, often or always)**	0 (0%)	27 (16%)	178 (29%)	343 (53%)	< 0.0001
**Loss or alteration of taste (%, often or always)**	1 (5%)	28 (16%)	159 (26%)	283 (44%)	< 0.0001
**Bad mouth breath (%, often or always)**	2 (11%)	30 (18%)	133 (21%)	212 (33%)	< 0.0001
**Gastroesophageal reflux (%, often or always)**	4 (21%)	62 (36%)	281 (45%)	323 (50%)	0.0001

#### Effects of xerostomia on the quality of life

Subjects experiencing dry mouth reported a poorer quality of life and were more concerned by feelings of oral discomfort, especially for “swallowing” and “talking,” interrupted sleep, discomfort in social life, and worries about the evolution of their mouth and the cost of dental care (Table 5).

**Table 5:** Evaluation of the association between dry mouth and quality of life. Participants were deemed to have oral dryness if they answered ‘always’ or ‘quite often’ to the question that explored the frequency of experiencing of dry mouth

		Without oral dryness (%) *N* = 190	With oral dryness (%) *N* = 1268	***p***-value (Pearson’s Chi-squared test)
**Feelings of oral discomfort**	Never	41 (21.58)	41 (3.23)	< 0.0001
	Sometimes	108 (56.84)	228 (17.98)	
	Quite often	28 (14.74)	566 (44.64)	
	Always	13 (6.84)	433 (34.15)	
**Interrupted sleep**	Never	80 (42.11)	125 (9.86)	< 0.0001
	Sometimes	86 (45.26)	461 (36.36)	
	Quite often	16 (8.42)	420 (33.12)	
	Always	8 (4.21)	262 (20.66)	
**Discomfort in social life**	Not at all	77 (40.53)	84 (6.62)	< 0.0001
	A little bit	100 (52.63)	571 (45.03)	
	A lot	12 (6.32)	451 (35.57)	
	Very much	1 (0.53)	162 (12.78)	
**Worries about the evolution of mouth problems**	Not at all	48 (25.26)	87 (6.86)	< 0.0001
	A little bit	98 (51.58)	503 (39.67)	
	A lot	33 (17.37)	469 (36.99)	
	Very much	11 (5.79)	209 (16.48)	
**Worries about the cost of dental care**	Not at all	55 (28.95)	188 (14.83)	< 0.0001
	A little bit	70 (36.84)	427 (33.68)	
	A lot	42 (22.11)	383 (30.21)	
	Very much	23 (12.11)	270 (21.29)	
**Alteration of the quality of life**	Not at all	58 (30.53)	53 (4.18)	< 0.0001
	A little bit	105 (55.26)	519 (40.93)	
	A lot	19 (10)	483 (38.09)	
	Very much	8 (4.21)	213 (16.8)	

Participants were deemed to have oral dryness if they answered ‘always’ or ‘quite often’ to the question that explored the frequency of experiencing of dry mouth

Dose-response relationships were found between the frequency of dry mouth and each of the quality-of-life dimensions (Table 6): the drier the mouth, the more the various dimensions of the patients’ quality of life were affected.

**Table 6:** Association between the intensity of experienced dry mouth and the dimensions of the quality of life

	Frequency of dry mouth				***p*** for trend (Cochran Armitage Chi-squared test)
	Never ***N*** = 19	Sometimes ***N*** = 171	Quite often ***N*** = 623	Always ***N*** = 645	
**Feelings of oral discomfort (%, often or always)**	1 (5%)	40 (23%)	417 (67%)	582 (90%)	< 0.0001
**Interrupted sleep (%, quite often or always)**	1 (5%)	23 (13%)	279 (45%)	403 (62%)	< 0.0001
**Discomfort in social life (%, a lot or enormously)**	1 (5%)	12 (7%)	188 (30%)	425 (66%)	< 0.0001
**Worries about the evolution of mouth problems (%, a lot or enormously)**	3 (16%)	41 (24%)	259 (42%)	419 (65%)	< 0.0001
**Worries about the cost of dental care (%, a lot or enormously)**	4 (21%)	61 (36%)	292 (47%)	361 (56%)	< 0.0001
**Alteration of the quality of life (%, a lot or enormously)**	1 (5%)	26 (15%)	229 (37%)	467 (72%)	< 0.0001

#### Efficiency of treatments for dry mouth

Most patients (62.68%) did not receive any treatment for dry mouth, regardless of the nature of the syndrome, because they were not recommended (data not shown). For the others (544 subjects), only pilocarpine was significantly associated with an improvement in the sense of dry mouth (22.19% reported no improvement in the feeling of dry a mouth versus 50.83% who reported an improvement, *p* <  0.0001, data not shown), whereas saliva substitutes were significantly associated with no improvement (84.44% reported no improvement in the feeling of dry a mouth versus 58.26% who reported an improvement, *p* <  0.0001, data not shown).

### Study of the role of the dentist in the detection and management of Sjögren’s syndrome

For this aspect, we analyzed only the responses received from patients with primary or associated Sjögren syndrome (1306 respondents). Our results suggest that most patients did not report their dry mouth to their dentist prior to being diagnosed with Sjögren syndrome (61.56% of patients). Indeed, only 270 participants who experienced dry mouth reported it to their dentist before having an etiological diagnosis. Among them, the dentist investigated the cause of dry mouth (16.7%) and evoked Sjogren’s syndrome (16.4%) by looking for other manifestations of Sjögren’s (7.04%) and/or referred patients to a medical team in most cases (25.19%) (Table 7).

**Table 7:** Dentist’s management of Sjögren’s syndrome after being informed by the patient. The number of patients who answered "Yes" to the question "Before I was diagnosed with Sjögren's syndrome, I told my dentist about my dry mouth sensation" and who had primary or associated Sjögren's syndrome was 270. The table focuses on this population

***N*** = 270	Yes
**My dentist has investigated possible causes of dry mouth**	45 (16.7%)
**My dentist looked for possible manifestations of Sjögren syndrome**	19 (7.04%)
**My dentist mentioned Sjögren syndrome**	36 (16.4%)
**My dentist referred me to a doctor or other healthcare professional**	68 (25.19%)

The number of patients who answered “Yes” to the question “Before I was diagnosed with Sjögren’s syndrome, I told my dentist about my dry mouth sensation” and who had primary or associated Sjögren’s syndrome was 270. The table focuses on this population

Patients with Sjögren’s syndrome declared having received little information or advice in managing the risk of caries. For example, only 26.11% declared having received a prescription for specific oral hygiene products, such as fluoridated toothpaste, a figure that fell to 4.29% for a prescription for fluoridation trays. In terms of oral hydration, less than 10% of respondents reported receiving a prescription for oral fluid substitutes (9.1%) or daily life hydration counseling (9.19%) (Table 8).

**Table 8:** Analysis of dentist’s recommendations. The number of patients with primary or associated Sjögren syndrome was 1,306. The table focuses on this population

***N*** = 1306	Yes
**My dentist has prescribed specific oral hygiene products (ex: fluoride-rich toothpaste)**	341 (26.11%)
**My dentist has prescribed fluoridation trays**	56 (4.29%)
**My dentist has prescribed saliva substitutes**	118 (9.1%)
**My dentist has prescribed daily living tips (ex: hydration)**	120 (9.19%)

The number of patients with primary or associated Sjögren syndrome was 1306. The table focuses on this population

These results are coherent with the fact that a large number of patients with Sjögren’s syndrome (*n* = 58%) had not been informed about the oral risks of the syndrome by their dentist and sought information themselves or from their physician (data not shown).

## Discussion

A dry syndrome consists of a set of symptoms and clinical manifestations resulting from a reduction in secretion from various mucous membranes of the body. Sjögren’s syndrome is an autoimmune disease characterized by involvement of the entire exocrine glandular system that can be isolated or associated with another autoimmune disease. The main feature of such syndromes is persistent dryness of the eyes and mouth. One aim of this survey was to assess the experience of patients suffering from dry mouth in these contexts. This study was carried out with the active collaboration of the AFGS patient association. It can be assumed that most of the respondents were members of the association. Indeed, most of the respondents were women over 50 (consistent with the prevalence of Sjogren’s syndrome) and were mainly concerned with primary Sjogren’s syndrome. According to the AFGS, patients with associated Sjögren’s are more likely to join an association relating to their associated autoimmune disease.

Based on the patient reports and in accordance with the literature, patients with Sjögren’s syndrome were mostly concerned about caries [10], tooth wear [11], candidiasis [12], loss and alteration of taste [13, 14], and orofacial pain, which could be neuropathic in origin, such as stomatodynia and glossodynia. These alterations may be directly due to the hyposalivation found in most cases of dry mouth and Sjogren’s syndrome, as well as the acidic environment due to gastroesophageal reflux disease declared here and in the literature [15]. One of the limitations of this study may have been the ability of the patients to identify and report their oral health problems. We aimed to avoid this situation by working with AFGS patients, who corrected the questions to improve patient understanding and cover all the areas expected by them. Moreover, many studies have shown that the ability of patients to provide reliable information on the state of their oral health depends on timing, age, and country/culture [16]. However, it is the most “important” events, such as the declaration of the loss of a tooth, that are the most reliable. Thus, observational studies are still essential to validate the information declared by patients. For example, concerning “aphthous” lesions, this item mostly relates to the perception of a modification of the oral mucosa, corresponding to a wide range of lesions or diseases of the oral mucosa, some of which may be favored by the autoimmune component of Sjögren’s disease [17]. Identifying the nature of oral mucosal lesions requires clinical studies but this is challenging due to their rarity in the context of a rare disease.

The questions proposed to assess the oral quality of life of patients were developed by concertation between clinical researchers and patients. They were selected based on the OHIP-14 Xerostomia Inventory (dry mouth analysis tool) [18] and clinical experience as a clinician and patient. We found a strong impact of dry mouth on the quality of life, in accordance with the literature [19], in terms of social life (discomfort in social life), functional limitations (discomfort in the mouth, interrupted sleep), and psychological stress (concern about the evolution of the mouth and the costs of care). These results can be mainly explained by hyposalivation, as most of the patients using or having used dry mouth treatments did not experience any improvement in their dry mouth, with the exception of those using pilocarpine (as described in the literature -[20]).

The second aim of our study was to assess the role of the dentist in the detection and management of Sjögren’s syndrome. Most patients indicated that if they reported suffering from dry mouth, most dentists investigated the cause and, in particular, the existence of Sjögren’s syndrome. However, few patients told their dentists that they suffered from dry mouth, which also means that few dentists asked their patients about it, even if the signs of dryness may be visible, such as, for example, bullous or thick saliva. This is extremely unfortunate, because a recent questionnaire-based study revealed that evaluation of the existence of frequent or permanent xerostomia (found in 112 patients, corresponding to 8% of the population) allowed a medical team (dentists and possibly doctors) to make a diagnosis of dry mouth syndrome or Sjögren’s syndrome for eight and two patients respectively based on the measurement of unstimulated salivary flow, a simple and rapid procedure that does not require any specific equipment [21]. Moreover, our results raise questions about the actual role of dentists in the management of dry mouth. Indeed, patients reported having received few prescriptions to prevent dental caries, especially for highly fluoridated toothpaste or fluoride trays, for which the efficacy is proven and their prescription indicated for patients with dry mouth [22].

The duration of time required to diagnose Sjögren’s syndrome has been estimated to range from 35 to 120 months [23], and this timeframe is strongly correlated with the presence of dry mouth, a condition that significantly contributes to deteriorating oral health. Recent research, utilizing data from the Taiwan National Health Insurance Research Database Registry, reveals that individuals with Sjögren’s syndrome exhibit a notably higher utilization of annual outpatient dental services during the eight-year period leading up to their diagnosis [24]. Consequently, healthcare professionals have a crucial role to play in the early detection of Sjögren’s syndrome by considering it as a potential differential diagnosis for patients presenting with complaints related to xerostomia.

However, it is noteworthy that the diagnosis of pSS is predominantly carried out by rheumatologists rather than dentists [25]. This fact is substantiated by surveys conducted among dentists in Scotland and Germany, which indicate that dentists possess a commendable level of knowledge regarding the prevalence and etiology of dry mouth. Moreover, the researches demonstrate that younger general practitioners, still in their undergraduate phase of education, and dentists who have pursued postgraduate training exhibit even greater proficiency in this area. Dentists acknowledge the adverse impact of dry mouth on patients’ quality of life and recognize the significance of providing tailored dental prophylactic measures. Nevertheless, there appears to be a gap in their familiarity with the diagnostic processes related to dry mouth [26, 27]. Accurate diagnosis necessitates an understanding of the clinical manifestations of a wide spectrum of medical conditions, a domain in which dentists may receive less extensive training and, subsequently, exhibit reduced confidence. Additionally, it should be acknowledged that pSS is a relatively rare disorder, affording dentists fewer opportunities to encounter and diagnose it in their practice, thereby limiting their expertise in this particular field [21].

Our results must be interpreted with caution due to several biases resulting from the use of a self-administered questionnaire. First, the survey was not restricted to members of the AFGS. Therefore, some supporters (not patients) or patients for whom the diagnosis of the disease is being investigated could have responded to the survey. We hoped to avoid this situation by opening the questionnaire with a patient declaration of primary Sjögren’s, secondary Sjögren’s, or Sicca’s syndrome (no other choice possible). However, this is a self-report, and we did not ask about confirmation of a medical diagnosis by a specialist. In other words, as dry mouth is not always directly linked to Sjögren’s syndrome [28], the people concerned may suffer from other conditions that cause this symptom. A second limitation was that no explanation was offered to patients regarding the terminology “associated Sjögren’s” and it is possible that this may have caused confusion for some patients. Nonetheless, the distribution of responses concerning the symptoms (dry mouth, dry eyes, fatigue, articular pain) reported by the patients is concordant with the categories of primary Sjögren’s, associated Sjögren’s, and Dry Syndrome. A third limitation was that we were subject to measurement bias, i.e., we may not have measured what we wanted to measure because of the way the questions were formulated/interpreted (e.g., history of dental caries), as well as recall bias (difficulty remembering past events) and subjective bias (desire to correspond to what is being investigated). We anticipated these biases when preparing the survey, as we tested the questionnaire on a panel of patients involved in the AFGS. Nevertheless, although a self-administered questionnaire may not be as well completed as a questionnaire administered in face-to-face interviews or provide results as reliable as a clinical study, it allows the inclusion of a greater number of participants. Indeed, the strength of this survey was the high participation rate, increasing the statistical power of this work and highlighting the interest of the patients in oral health.

## Conclusion

This survey reinforces the data in the literature on the impact of dry mouth on oral health and highlights the need for dentists to promote screening and prevention of these diseases.

### Supplementary Information

**Additional file 1.**

## Data Availability

The datasets used during the current study are available from the corresponding author on reasonable request.
