# Supplementary material for: The experience of dry mouth and screening for Sjogren’s syndrome by the dentist: patient-reported experiences
Source: BMC Oral Health. 2023 Dec 15;23:1010. doi: 10.1186/s12903-023-03727-z (PMC10724976; doi:10.1186/s12903-023-03727-z)
Supplement: Supplementary file 1 — Additional file 1. [file 12903_2023_3727_MOESM1_ESM.docx]

**ANNEX 1 :** “Survey questionnaire”

**PART 1: Characteristics of the population:**

1. **I am:**

□ a man

□ a women

1. **I am:**

□ Less than 40 years old

□ 40 to 50 years old

□ 50 to 60 years old

□ Over 60 years old

1. **I am affected by:**

□ Primary Sjögren’s Syndrome

□ Secondary/ associated Sjögren’s Syndrome

□ Dry Syndrome (no Sjögren syndrome diagnosed)

1. **Among these four symptoms, the one that affects my quality of life the most is:**

□ Eye dryness

□ Mouth dryness

□ Articular and muscular pain

□ Asthenia

**PART 2: Dry mouth characteristics and remedies:**

1. **My mouth feels dry:**

□ Never

□ Sometimes

□ Quite often

□ Always

1. **I take treatments for dry mouth:**

□ Yes

□ No

**6Bis. If so, I:**

□ Take saliva substitutes (Novasial®,Aequasyal®, Artisial  spray®, Elgydium spray®, gel GUM  Hydral®, Bioxtra gel®, ...)

□ Take homeopathic treatments (tincture of Jaborandi, granules Nux  Moschata, …)

□ Take treatment with essential oils (Sweet Traamond, Eucalyptus, gomenol, coconut, tea tree, borage, …)

□ Take pilocarpine (Salagen®…)

□ Chew-gum

**6Ter. If so, I feel an improvement of dry mouth:**

□ Not at all

□ A little bit

□ Some

□ A lot

**6Quater. If not, I don't take treatments for dry mouth because:**

□ they are not effective

□ the side effects are too strong

□ they are too expensive

□ they haven't been recommended/prescribed to me or I don't know how to choose them.

**PART 3: Characteristics of oral problems:**

1. **I suffer from oral problems:**

□ Yes

□ No

**7Bis. If so, of what nature are they:**

□ tooth decay

□ dental wear (crumbling teeth...)

□ gingiva (bleeding, pain, swollen gums, loosening of the teeth, tooth mobility)

□ early tooth care failures (frequent loss of restorations, need to have care repeated every year....)

□ difficulties in wearing removable prostheses (partial or total dentures)

□ implant placement failures

1. **I suffer from mouth ulcers:**

□ Never

□ Sometimes

□ Quite often

□ Always

1. **I suffer from oral candidiasis:**

□ Never

□ Sometimes

□ Quite often

□ Always

1. **I suffer from oral pain or burning:**

□ Never

□ Sometimes

□ Quite often

□ Always

1. **I suffer from the loss or alteration of taste:**

□ Never

□ Sometimes

□ Quite often

□ Always

1. **I suffer from bad breath:**

□ Never

□ Sometimes

□ Quite often

□ Always

1. **I suffer from gastro-esophageal reflux:**

□ Never

□ Sometimes

□ Quite often

□ Always

**PART 4: Effects of dry mouth and its consequences on the quality of life:**

1. **Feeling of oral discomfort:**

□ Never

□ Sometimes

□ Quite often

□ Always

**14bis. (if sometimes - quite often - always ticked). In this case, I find it difficult to:**

□ chew food

□ swallow (I drink water to swallow)

□ speaking (speech defects/pronunciation)

1. **My dry mouth interrupts my sleep several times a night:**

□ Never

□ Sometimes

□ Quite often

□ Always

1. **My dry mouth interferes with my social life:**

□ Not at all

□ A little bit

□ A lot

□ Very much

1. **I'm worried about the future of my mouth**:

□ Not at all

□ A little bit

□ A lot

□ Very much

1. **I'm worried about the cost of dental treatment:**

□ Not at all

□ A little bit

□ A lot

□ Very much

1. **My dry mouth (and its consequences) affects my quality of life:**

□ Not at all

□ A little bit

□ A lot

□ Very much

**PART 5: Assessment of the role of the dentist in screening for Sjögren's syndrome:**

Please note: Questions/section to be opened only if the patient has ticked primary or associated Sjögren’s syndrome in question 3.

In this section, we want to assess whether your dentist was involved in the diagnosis of Sjögren's syndrome. We are therefore referring to a period prior to the diagnosis.

1. **Before I was diagnosed with Sjögren's syndrome, I told my dentist about my sensation of dry mouth:**

□ Yes

□ No

□ I don't know anymore

**20BIS. If so, my dentist:**

□ investigated possible causes of dry mouth (diabetes, tobacco...)

□ looked for possible manifestations of Sjögren syndrome (episode of parotitis, other

dryness....)

□ mentioned Sjögren syndrome

□ referred me to a doctor or other healthcare professional

□ I don't know anymore

1. **My dentist played a role in the diagnosis of Sjögren's syndrome:**

□ Yes

□ No

1. **Before I was diagnosed with Sjögren's syndrome, my dentist pointed out that I had more oral problems than the average of his patients:**

□ Yes

□ No

**PART 6: Dentist's management of Sjögren's syndrome:**

Please Note: Questions/section to be opened only if the patient has ticked either primary or secondary Sjören’s syndrome in question 3.

In this section, we would like to assess whether your dentist has suggested any special management for Sjögren's syndrome. We therefore refer to the period following diagnosis.

1. **I have informed my dentist that I have Sjögren's Syndrome:**

□ Yes

□ No

**23Bis. If yes, my dentist knew about Sjögren's syndrome:**

□ Yes

□ No

□ I don't know

**23Ter. If yes, my/my dentist looked for information about Sjögren's syndrome:**

□ Yes

□ No

□ I don't know

1. **My dentist has informed me of the oral risks associated with Sjögren's syndrome (increased risk of caries, wear and tear, candidiasis...):**

□ Yes

□ No

**24Bis. If no, I received this information from:**

□ a doctor (rheumatologist, internist, general practitioner....)

□ a stomatologist

□ myself (I found them)

□ I did not receive information.

1. **My dentist prescribed:**

□ Brushing equipment (manual toothbrush with soft bristles, electric, interdental brushes...)

□ Brushing technique

□ Specific oral hygiene products (ex: fluoride-rich toothpaste)

□ Fluoridation trays

□ Saliva substitutes

□ Daily living tips (hydration)

□ No prescription

1. **On the advice of my dentist, I consult him/her for a follow-up:**

□ no advice given

□ less than once a year

□ 1 time a year

□ 2 times a year

□ more than 2 times a year

1. **I am followed up for my teeth by:**

□ a general dentist

□ a dentist in a dispensary

□ a dentist in a mutualist center

□ a dentist in a hospital
